# Earlier Menarche Is Associated with Lower Insulin Sensitivity and Increased Adiposity in Young Adult Women

**DOI:** 10.1371/journal.pone.0128427

**Published:** 2015-06-10

**Authors:** Dyanne A. Wilson, José G. B. Derraik, Deborah L. Rowe, Paul L. Hofman, Wayne S. Cutfield

**Affiliations:** 1 Liggins Institute, University of Auckland, Auckland, New Zealand; 2 School of Nursing, Faculty of Medical and Health Sciences, Auckland, University of Auckland; Faculty of Biology, SPAIN

## Abstract

**Objective:**

We aimed to assess whether age at menarche was associated with insulin sensitivity in young adult women.

**Methods:**

We studied 54 healthy young women aged 20–30 years. Participants were grouped according to age at menarche: Early (≤11.0 years; n=13), Average (>12.0 and ≤13.0 years; n=28), and Late (≥14.0 years, n=13). Primary outcome was insulin sensitivity measured using intravenous glucose tolerance tests and Bergman’s minimal model. Body composition was assessed using whole-body dual-energy X-ray absorptiometry.

**Results:**

Earlier menarche was associated with lower insulin sensitivity (p=0.015). There was also a continuous increase in adiposity with younger age at menarche, which was associated with increased weight (p=0.001), BMI (p=0.002), total body fat (p=0.049), and truncal fat (p=0.020). Stratified analyses showed that insulin sensitivity in Early women (5.5 x10^-4^·min^-1^(mU/l)) was lower than in Average (8.0 x10^-4^·min^-1^(mU/l), p=0.021) and Late (8.6 x10^-4^·min^-1^(mU/l), p=0.033) groups. Early women (weight=66.1 kg; BMI=24.1 kg/m^2^) were considerably heavier and fatter than Average (59.0 kg, p=0.004; 21.4 kg/m^2^, p=0.002) and Late (57.0 kg, p=0.001; 20.8 kg/m^2^, p=0.0009) women.

**Conclusions:**

Early menarche is associated with lower insulin sensitivity and increased adiposity in young adulthood, potentially increasing the risk of type 2 diabetes and the metabolic syndrome later in life.

## Introduction

The onset and timing of puberty in humans are not fully understood, but are dependent on numerous factors, including genetic variability, energy balance, brain structure, multiple neuroendocrine pathways, and hormonal profiles. Nonetheless, it is acknowledged that successful reproduction requires suitable energy stores to support the associated physiological functions [1]. Therefore, puberty is "metabolically gated" to prevent fertility in conditions of energy insufficiency [2], so that metabolic conditions and the amount of energy reserves play an important role modulating puberty timing [1,2]. Peptides from the digestive tract such as ghrelin (i.e. energy intake) and adipose tissue such as leptin (i.e. energy storage) inform the central nervous system about the individual's current metabolic status [1]. These peptides are important modulators of the gonadotropic axis that are capable of disturbing pubertal onset or progression [1].

Leptin has been shown to provide important signals of energy sufficiency, working at the level of the hypothalamus to modulate the function of the gonadotropin-releasing hormone (GnRH) neuronal system [2,3]. There is evidence that insulin, alongside leptin, also plays a role in the modulation of GnRH neurons and reproductive development [4,5]. An experimental model *in vivo* has shown that increasing insulin concentrations stimulated luteinizing hormone secretion, while an *in vitro* model showed a direct dose-dependent stimulation of GnRH secretion by insulin [6]. As a result, insulin sensitivity during childhood may also be important in the initiation of and progression through puberty.

Adolescents with anorexia nervosa (who are nutritionally deficient) and those with chronic illness (who have a combination of nutritional, inflammatory, and therapeutic compromises) have delayed puberty and enhanced insulin sensitivity [7,8]. Children with constitutional delay of growth and development are also more insulin sensitive, even after controlling for adiposity [9]. In contrast, obese children have an earlier onset of puberty [10] and lower insulin sensitivity [11], while children born prematurely or small-for-gestational-age (SGA) (both associated with decreased insulin sensitivity) have an increased risk of premature adrenarche and early puberty [12]. Insulin sensitivity seems to play an important physiological role during puberty, when a marked reduction in insulin sensitivity occurs coinciding with Tanner 2 stage of development [13]. This reduction in insulin sensitivity is proposed to be important to pubertal development, as it leads to increased circulation of growth factors and promotes protein (muscle) production [14].

We therefore hypothesised that the timing of puberty would affect insulin sensitivity in young adult women; i.e. early puberty would be associated with lower insulin sensitivity and late puberty with increased insulin sensitivity. Thus, we aimed to determine whether age at menarche was associated with insulin sensitivity in early adulthood.

## Methods

### Ethics approval

This study was approved by the Northern X Regional Ethics Committee (Ministry of Health, New Zealand). All procedures followed were in accordance with the ethical standards of the responsible committees on human experimentation and with the Declaration of Helsinki. Both written and verbal informed consents were obtained from all participants.

### Participants

Healthy women aged 20–30 years were recruited to this study through advertising among staff and students at University of Auckland campuses (Auckland, New Zealand). All women were childless, not pregnant, and non-smokers, had no history of drug or alcohol abuse, and had not used oral contraceptives for the preceding 6 months. Exclusion criteria were evidence of chronic illness (including behavioral disorders such as attention deficit hyperactivity disorder), obesity (BMI ≥30.0 kg/m^2^), chromosomal or syndromal diagnosis, conception by means of *in vitro* fertilization, use of medication known to influence insulin sensitivity, having first-degree relatives with type 2 diabetes mellitus or the metabolic syndrome, and present or past history of an eating disorder. Those born SGA, preterm (<37 weeks of gestation), or after twin pregnancy were also excluded, as these conditions are associated with reduced insulin sensitivity. All potential participants were screened for type 1 diabetes antibodies, and those with the presence of islet cell antibodies [glutamic acid decarboxylase antibody (antiGAD) or islet antigen 2 antibody (IA2)] were also excluded.

### Study design

Age at menarche was established based on an individual's recall. A number of previous studies have shown that menarcheal age recall is highly correlated with the actual recorded age [15,16]. A study by Must et al. on over 400 women showed that, on average, menarcheal age recall after 30 years was only 0.08 years earlier (95% confidence interval: -0.18–0.01 years) than the actual age at menarche [16]. In comparison, the time elapsed between actual menarcheal age and our study was much shorter: 9.9 ± 2.9 years (range 3.2–19.0 years).

Apart from examining linear associations between age at menarche and study outcomes, participants were divided into three groups according to their age at menarche: Early (≤11.0 years), Average (>12.0 and ≤13.0 years), and Late (≥14.0 years). These thresholds were chosen to approximate the distribution of age at menarche in the Non-Hispanic white population in the USA [17], as these data would more closely reflect New Zealand Europeans who would very likely constitute the bulk of our cohort. Thus, our Early group fell below the 10^th^ percentile for age at menarche (11.32 years), the Average group between the 25^th^ and 75^th^ percentile (11.9–13.2 years), and the Late group above the 90^th^ percentile (13.78 years) [17]. Importantly, we also established a one-year gap between menarcheal age groups not only to account for potential recall inaccuracies, but also to ensure the group division was applicable to all ethnicities.

### Clinical assessments

Assessments were carried out after a 12-hour overnight fast at the Maurice & Agnes Paykel Clinical Research Unit (Liggins Institute, University of Auckland). All women were assessed during the early follicular phase of their menstrual cycle (d5–10), as studies have suggested that there are variations in insulin sensitivity during the normal menstrual cycle [18,19].

Clinical history was obtained, including menstrual history, use of oral contraception, exercise history, and assessment of other risk factors for insulin resistance. A physical examination was conducted to exclude evidence of chronic illness, including checking for acanthosis nigricans. Ethnicity was recorded by self-report using a priority system, such that if multiple ethnicities were selected, the patient was assigned to a single ethnicity, following a hierarchical classification [20].

### Primary outcome

Insulin sensitivity was assessed using a 90-minute modified frequently sampled intravenous glucose test (FSIGT), modified with insulin, and analysed using Bergman’s minimal model software [21]. Three baseline samples were drawn at -20, -10, and 0 minutes. A 25% dextrose infusion (at 0.3 g/kg) started at 0 minute and lasted for one minute. Blood samples were drawn at 2, 3, 4, 5, 6, 8, 10, 12, 14, 16, and 19 minutes. Insulin (0.015 units/kg) was then intravenously administered as a bolus at 20 minutes, and further samples were drawn at 22, 23, 24, 25, 27, 30, 35, 40, 45, 50, 60, 70, 80, and 90 minutes.

### Secondary outcomes

Other parameters associated with glucose homeostasis were the acute insulin response (the integrated insulin levels over the first 10 minutes of the FSIGT following the initial intravenous glucose bolus), glucose effectiveness (an estimation of glucose-mediated glucose uptake), and disposition index (the product of insulin sensitivity x acute insulin response). Participants' heights were measured using a Harpenden stadiometer to the nearest 1 mm. Weight and body composition data were obtained using whole-body dual-energy X-ray absorptiometry (DXA, Lunar Prodigy 2000, General Electric, Madison, WI, USA). Parameters of interest were: total body fat, fat-free mass, and truncal fat percentages, as well as bone mineral density.

### Assays

Plasma glucose was measured with a Hitachi 902 Automatic Analyzer (Hitachi Scientific Instruments, Tokyo, Japan) with an inter-assay coefficient variation of 1.8%. Plasma insulin was determined with Abbott’s IMX Microparticle Enzyme Immunoassay (Abbott, Seoul, Korea), with an inter-assay coefficient variation of 4.9%.

### Statistical analyses

Stratified analyses were carried out using multiple linear regression models to assess primary and secondary outcomes. Confounding factors were controlled for in the analyses as required, depending on the outcome response of interest: for insulin sensitivity and outcomes associated with glucose homeostasis—age and total body fat percentage were included; and for body composition—age and ethnicity. Possible associations were also investigated with menarcheal age as a continuous variable. All multivariate analyses were performed in SAS version 9.3 (SAS Institute Inc. Cary NC, USA). All statistical tests were two-tailed and maintained at a 5% significance level. Demographic data are presented as means ± standard deviations. Primary and secondary outcome data are presented as model-adjusted means (estimated marginal means adjusted for the confounding factors), with associated 95% confidence intervals. Parameters of glucose homeostasis were log-transformed to approximate normality. For linear associations, apart from p-values, the estimated mean change in the outcome response per year increase in age at menarche (β) is also provided.

## Results

We studied a total of 54 women aged 22.5 ± 2.3 years (range 20.1–30.0 years) and with a BMI of 22 ± 3 kg/m^2^ (range 18–29 kg/m^2^). There were 13 women in the Early, 28 in the Average, and 13 in Late groups, who were of similar age (22.7 ± 2.9, 22.6 ± 2.0, and 22.0 ± 2.3 years, respectively). Overall, 65% of participants were of New Zealand European ethnicity (with the remainder including Asians, Indians, and Pacific Islanders), and there were no statistically significant differences in ethnic composition between groups (p = 0.19).

### Linear associations

Insulin sensitivity varied according to menarcheal age, with earlier menarche associated with a reduction in insulin sensitivity (r^2^ = 0.13; β = 0.12; p = 0.015). Accordingly, earlier menarche was associated with greater acute insulin response (r^2^ = 0.17; β = -0.19; p = 0.002). In relation to anthropometry, earlier menarche was associated with an increase in adiposity, including increased weight (r^2^ = 0.18; β = -2.07; p = 0.001), BMI (r^2^ = 0.19; β = -0.73; p = 0.002), total body fat (r^2^ = 0.07; β = -1.04; p = 0.049), and truncal fat (r^2^ = 0.10; β = -1.74; p = 0.020).

### Stratified analyses

**Fig 1:**
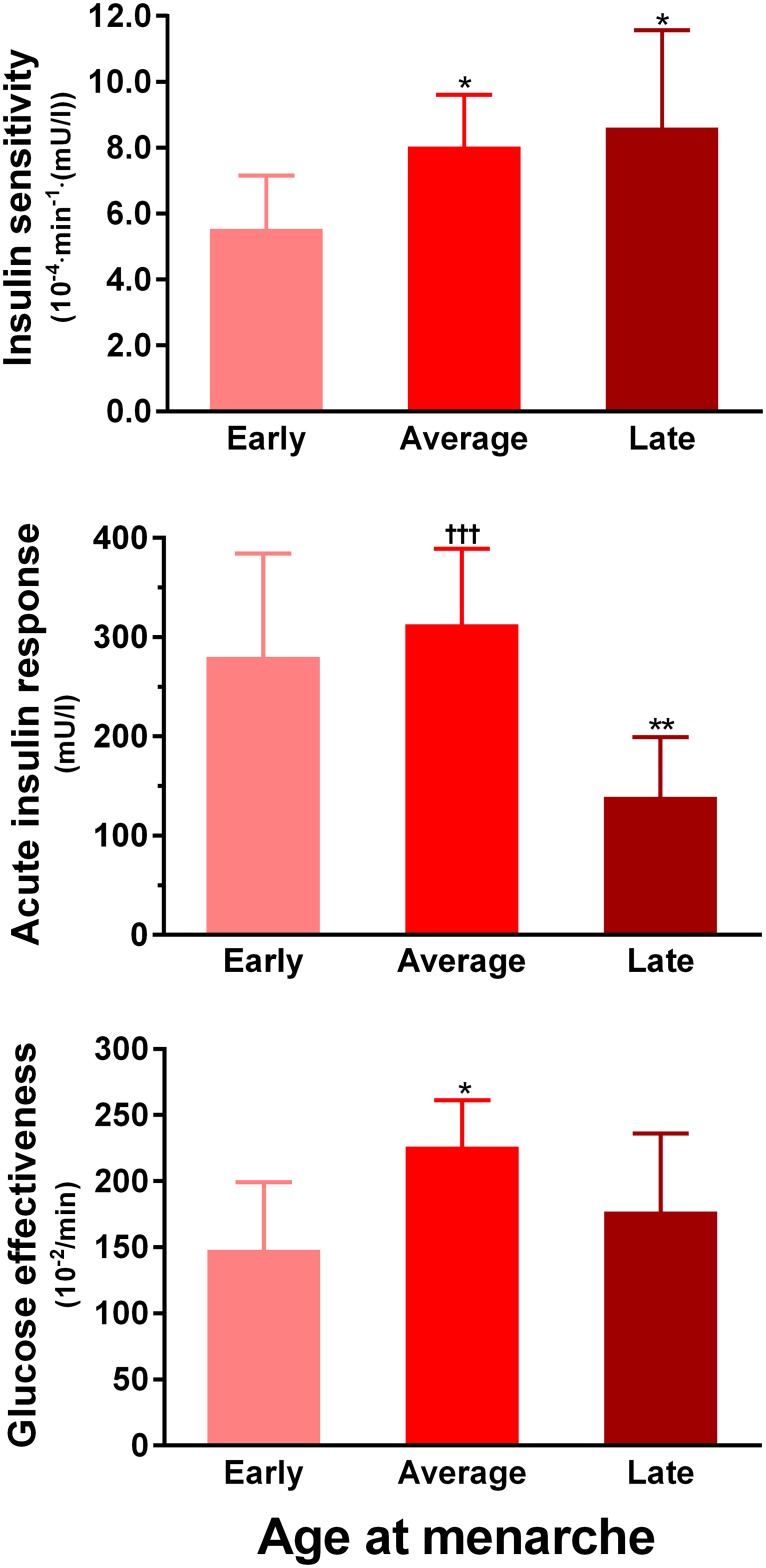
Insulin sensitivity and other parameters of glucose homeostasis according to menarcheal age groups: Early (n = 13), Average (n = 28), and Late (n = 13). Data are adjusted means from multivariate models with respective 95% confidence intervals. *p<0.05 and **p<0.01 vs Early group; †††p<0.001 vs Late group.

Insulin sensitivity in Early women (5.5 x10^-4^·min^-1^(mU/l)) was 31% lower than in Average (8.0 x10^-4^·min^-1^(mU/l), p = 0.021) and 36% lower than in Late (8.6 x10^-4^·min^-1^(mU/l), p = 0.033) groups (Fig 1). The acute insulin response was higher in both Early (280 mU/l, p = 0.007) and Average (313 mU/l, p = 0.0004) groups compared to Late women (139 mU/l) (Fig 1). Early women also had lower glucose effectiveness (148 vs 226 x10^-2^/min, p = 0.016; Fig 1) and lower disposition index (1555 vs 2515 mg/dl, p = 0.009) than Average women, who in turn had greater disposition index than Late women (2515 vs 1198 mg/dl, p = 0.0004). There were however, no differences in fasting glucose or insulin concentrations (Table 1), which are less precise indices of glucose regulation. Importantly, all observed differences in glucose homeostasis were adjusted for total body fat percentage and age.

**Table 1:** Demographic data on studied women according to age at menarche.

		Early	Average	Late
**n**		13	28	13
**Demography**	Age (years)	22.7 ± 2.9	22.6 ± 2.0	22.0 ± 2.3
	Ethnicity (New Zealand European)	47%	75%	62%
**Anthropometry**	Height (cm)	167 (163–171)	163 (160–166)	164 (160–168)
	Weight (kg)	66 (62–70)	59 (63–56)**	57 (61–53)**
	BMI (kg/m^2^)	24 (23–26)	21 (20–22)**	21 (19–22)***
	Total body fat (%)	33 (30–37)	29 (26–31)*	29 (25–33)
	Truncal fat (%)	36 (31–40)	31 (26–36)	29 (23–34)*
	Fat-free mass (%)	66 (63–70)	71 (68–73)	71 (66–75)
	Waist-to-hip ratio	0.73 (0.70–0.75)	0.73 (0.71–0.75)	0.73 (0.70–0.75)
	Bone mineral density (g/cm^2^)	1.19 (1.15–1.23)	1.16 (1.13–1.19)	1.14 (1.10–1.19)
	L2–L4 bone mineral density (g/cm^2^)	1.22 (1.15–1.30)	1.20 (1.15–1.26)	1.15 (1.08–1.23)
**Fasting blood parameters**	Glucose (mmol/l)	4.7 (4.5–5.0)	4.7 (4.5–4.8)	4.6 (4.4–4.9)
	Insulin (mU/l)	5.0 (3.9–6.4)	4.5 (3.8–5.3)	4.5 (3.5–5.8)

Age data are means and standard deviations; other data are adjusted means from multivariate models with respective 95% confidence intervals.

*p<0.05,

**p<0.01,

***p<0.001 vs Early group.

Early women were considerably heavier and of greater BMI than Average (p = 0.004 and p = 0.002, respectively) and Late (p = 0.001 and p = 0.0009, respectively) women (Table 1). Early women also had more total body fat than Average (p = 0.030) and more truncal fat than Late (p = 0.045) women (Table 1). As a result, there were considerably more Early women overweight (BMI 25.0–29.9 kg/m^2^, 46%) than in both Average (7%, p = 0.007) and Late (8%, p = 0.033) groups. There were however, no differences in height, waist-to-hip ratio, or bone mineral density (Table 1).

## Discussion

This study shows that earlier menarche was associated with lower insulin sensitivity and higher insulin secretion. In addition, women who experienced earlier menarche were also heavier and had greater truncal adiposity. Notably, our findings on insulin sensitivity were not simply a result of increased adiposity, as total body fat percentage was adjusted for in the analyses. Importantly, all women were assessed during the early follicular phase of their menstrual cycle and none were on oral contraceptives for at least 6 months, in order to minimize the effects of varying oestrogen levels on insulin sensitivity [22,23].

To contextualize our findings, the magnitude of the difference in insulin sensitivity between Early and Late women (41%) was twice as large as the change seen with medications used to treat diabetes in adults, such as metformin (20%). Our observations corroborate previous studies showing younger menarcheal age to be associated with higher fasting insulin concentrations and increased insulin resistance as measured by HOMA-IR [24,25]. However, ours is the first study to actually measure insulin sensitivity using robust methods in association with menarcheal age. In addition, we showed changes in glucose regulation across the menarcheal age spectrum while total body fat was accounted for in our statistical models. Importantly, lower insulin sensitivity is associated with increased risk of developing type 2 diabetes mellitus, hypertension, coronary heart disease, stroke, and cancer in the long-term [26]. Thus, our finding are also in accordance with population studies that showed an increased risk of pre-diabetes, diabetes [27–29], and the metabolic syndrome [30] in association with earlier age at menarche.

We also observed a pronounced association of younger menarcheal age with increased adiposity in young adult women. Our data are consistent with several large investigations showing an association between earlier menarche and increased adiposity in adulthood [28,31–33]. However, as discussed by Ong et al. [34], the causal direction and mechanisms underpinning this association are unclear, as girls experiencing earlier menarche are more likely to be overweight even before the onset of puberty [33,35]. Increased childhood adiposity is associated with earlier puberty, particularly in girls, but may also be associated with more rapid pubertal progression [33,35,36], which in turn was shown to be associated with increased adiposity in young adulthood [37]. As a result, Must et al. proposed that in reality, it is the increased adiposity in childhood that leads to both earlier sexual maturation and greater adiposity in adulthood [35]. Nevertheless, the available evidence have not determined whether obesity drives earlier onset of puberty and insulin resistance, or whether insulin resistance occurs leading to obesity and earlier menarche.

The exact mechanisms regulating the timing of puberty are poorly understood. Association studies in twins suggest that 70–80% of the variation in pubertal timing may be explained by heritable factors [38]. Nonetheless, the timing of pubertal onset is likely a result of a complex interaction from a number of genetic and external factors, most of which have been discussed in depth by Parent et al. [38]. It is possible that signals associated with fat mass such as leptin and insulin-like growth factor I (IGF-I) may be involved in the regulation of pubertal onset [1,2,38].

Our findings are consistent with the existing literature indicating that insulin sensitivity or insulin levels play a role in pubertal onset [6,39]. During normal pubertal development, there is a reduction in insulin sensitivity that is mediated by increased growth hormone concentrations and a consequent increase in IGF-I levels [40]. We have also observed that pre-pubertal children with constitutional delay of growth and development were more insulin sensitive than normal children, which is consistent with our findings of a continuous association between age at menarche and insulin sensitivity [9]. In addition, support for a role for insulin sensitivity in pubertal onset has been shown by effects of an insulin sensitizer (metformin) in girls born SGA (who were insulin resistant), which delayed the onset of puberty, prolonged its duration, and reduced the proportion of fat mass [41,42]. Further, a recent study in rats has provided evidence that hyperinsulinaemia (a marker of insulin resistance) drives diet-induced obesity [43]. Therefore, it is possible that, in humans, greater insulin resistance/hyperinsulinaemia may lead to both increased adiposity and a premature pubertal onset. If this hypothesis is correct, it is important to understand the genetic, epigenetic, or environmental factors that are associated with these changes in insulin sensitivity in developmentally normal pre-pubertal children.

We acknowledge that our study was cross-sectional, so that it was not possible to determine whether increased adiposity led to a reduction in insulin sensitivity or vice-versa. Moreover, our stratified analyses grouped participants based on data for the USA and not New Zealand, and the relatively small number of participants in the Early and Late groups limit our ability to extrapolate our findings to the wider population. In addition, menarcheal age was obtained by participants' recall rather than actual recorded ages. However, this method is accurate [15,16], and our stratified analyses with a one-year gap between groups limited the impacts of potential recall errors.

In conclusion, our study provides direct evidence of altered insulin sensitivity in association with age at menarche, which has been previously inferred by studies using HOMA-IR [24,25,44]. Others have suggested that women who experienced early menarche may be at an increased risk of developing type 2 diabetes and other metabolic and cardiovascular diseases later in life [27–30]. However, the association between insulin sensitivity and age at menarche does not prove causality, and will require further longitudinal studies to better define this relationship. With the reduction in menarcheal age observed in recent decades throughout the world [45,46], our findings are of public health interest. Future research will need to help determine whether obesity or reduced insulin sensitivity is the principal driver for earlier pubertal onset.
